# Novel recombinant feline interferon carrying N-glycans with reduced allergy risk produced by a transgenic silkworm system

**DOI:** 10.1186/s12917-018-1584-z

**Published:** 2018-08-31

**Authors:** Sachi Minagawa, Yuzuru Nakaso, Masahiro Tomita, Takenori Igarashi, Yoshio Miura, Hideyo Yasuda, Satoshi Sekiguchi

**Affiliations:** 1 Innovation Center, Nippon Flour Mills Co., Ltd., 5-1-3 Midorigaoka, Atsugi, Kanagawa 243-0041 Japan; 2 Transgenic Silkworm Department, Immuno-Biological Laboratories Co., Ltd., 1091-1 Naka, Fujioka, Gunma 375-0005 Japan

**Keywords:** Feline interferon, Transgenic, Silkworm, N-glycan, Core α 1,3 fucosyl residues, Fucose, *Bombyx mori*

## Abstract

**Background:**

The generation of recombinant proteins for commercialisation must be cost-effective. Despite the cost-effective production of recombinant feline interferon (rFeIFN) by a baculovirus expression system, this rFeIFN carries insect-type N-glycans, with core α 1,3 fucosyl residues that act as potential allergens. An alternative method of production may yield recombinant glycoproteins with reduced antigenicity.

**Results:**

A cDNA clone encoding the fifteenth subtype of FeIFN-α (FeIFN-α15) was isolated from a Japanese domestic cat. This clone encoded a protein of 189 amino acids with a molecular mass of 21.1 kDa. The rFeIFN-α15 was expressed using a transgenic silkworm system, which was expected to yield an N-glycan structure with reduced antigenicity compared with the protein produced by the baculovirus system. The resulting rFeIFN-α15 accumulated in the sericin layer of silk fibres and was easily extracted and purified by column chromatography. The N-terminal amino acid sequence of purified rFeIFN-α15 was identical to the mature form of natural sequence. Moreover, its N-glycans did not include detectable core α 1,3 fucosyl residues. Its anti-vesicular stomatitis virus activity (2.6 × 10^8^ units/mg protein) was comparable to that of the baculovirus-expressed rFeIFN.

**Conclusions:**

The lower allergy risk of rFeIFN produced by the transgenic silkworm system than by the baculovirus expression system is due to the former lacking core α 1,3 fucosyl residues in its N-glycans. The rFeIFN-α15 produced by the transgenic silkworm system may be a prospective candidate for the next generation of rFeIFN in veterinary medicine.

## Background

Recombinant proteins generated for medical use should retain as much of their original structures as possible, including amino acid sequences and attached glycans. Any structural changes in these proteins may affect their bioactivity and/or bioavailability and may also increase the side effects associated with their use [1–4]. To avoid these shortcomings, despite their high production costs, most recombinant proteins used in humans are produced using mammalian cell lines.

A cost-effective method of producing recombinant proteins for veterinary medicine involves the use of the silkworm moth (*Bombyx mori*). Larvae or insect cell lines are infected with recombinant baculovirus [5–8], with the recombinant proteins accumulating in larval body fluid or the cultured cells. The post-translational modification pathways in insects such as silkworms are similar to those in mammalian cells, and various recombinant glycoproteins have been successfully produced. For example, commercially available recombinant FeIFN-ω (NCBI Number: E02521) was produced in silkworms using the baculovirus expression system [9–12].

Despite the cost advantages of the baculovirus expression system, there is concern with the structures of the N-glycans attached to the recombinant protein products. These are insect-type N-glycans, with a high percentage of core α 1,3 fucosyl residues. These core α 1,3 fucosyl residues have been found to induce allergic reactions when administered to mammals [13]. In fact, the rFeIFN-ω produced by this system is thought to contain core α 1,3 fucosylated N-glycans [14, 15]. Although the rFeIFN-ω has over 20 years of clinical experience, eliminating the insect type N-glycan is thought to be beneficial to improve the quality of veterinary medications in terms of potential allergenicity of core α 1,3 fucosyl residues [13, 16].

For this purpose in addition to the low cost feature of silkworm systems, we decided to use a transgenic system using silkworms [17–20]. In this system, foreign DNA carried on a vector plasmid is injected into silkworm eggs and is incorporated into the silkworm genome. Recombinant proteins synthesised in the middle silk gland (MSG) are secreted into the sericin layers of silk fibres of cocoons, allowing these proteins to be easily solubilised in aqueous buffer.

The N-glycan profile of recombinant glycoproteins produced by this transgenic silkworm system was assessed using recombinant mouse IgG [20]. These experiments showed that the N-glycans attached to the recombinant IgG did not include typical insect-type oligosaccharides, paucimannosidic glycans with core α 1,3 fucosyl residues [21–23]. Because the sericultural industry requires the production of large numbers of silkworm cocoons annually, the transgenic silkworm system may become a basic technology for the cost-effective production of recombinant proteins carrying non-allergic N-glycans.

Using a cDNA clone encoding a newly discovered FeIFN α subtype, FeIFN-α15, isolated from the blood of a Japanese domestic cat, this study evaluated the properties of rFeIFN produced by the transgenic silkworm system.

## Results

### Isolation of a clone of FeIFN

**Fig. 1:**
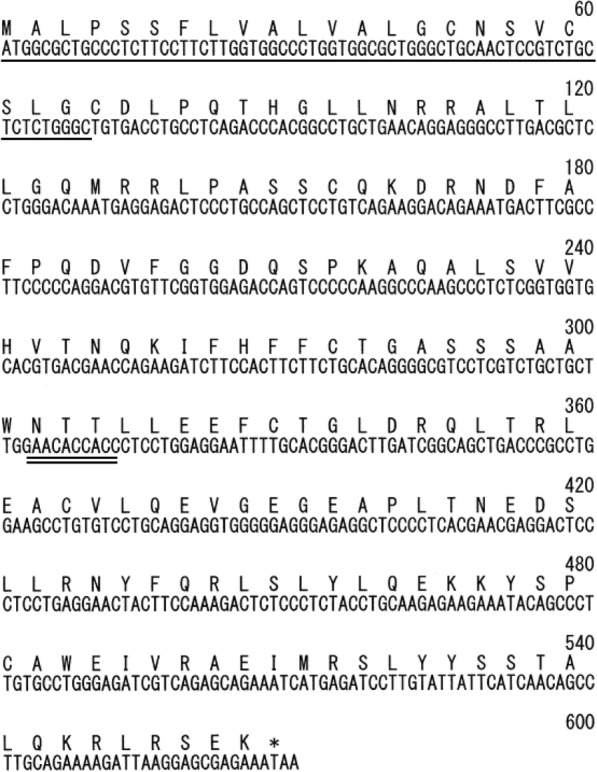
FeIFN-α15 cDNA and deduced amino acid sequences. Amino acids 1–23 (underlined) are thought to be a signal sequence. The double underline indicates a consensus site for asparagine-linked glycosylation

**Fig. 2:**
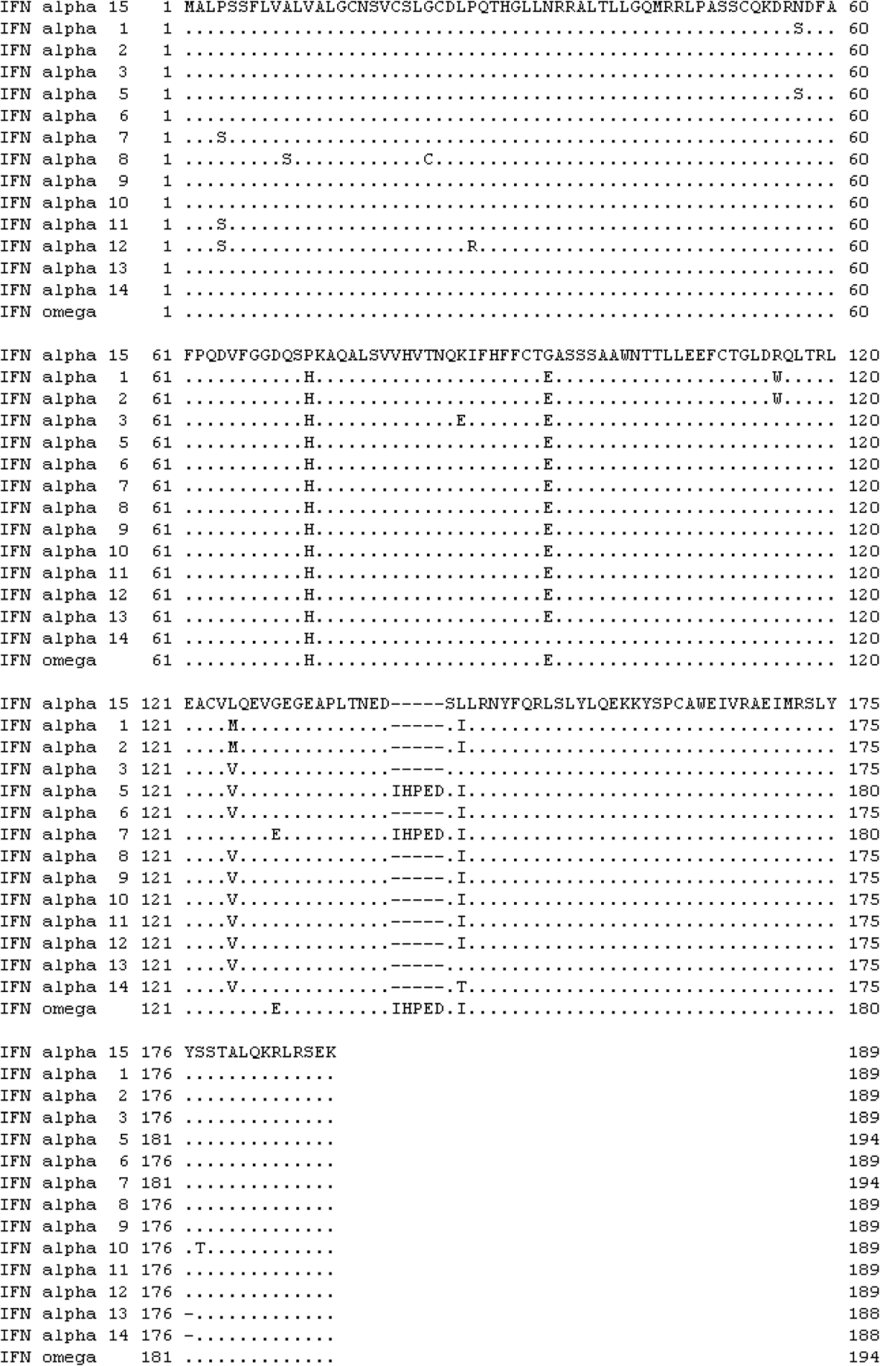
Homology of amino acid sequences of feline IFN alpha subtypes 1, 2, 3, 5, 6, 7, 8, 9, 10, 11, 12, 13, and 14 and IFN omega and IFN alpha subtype 15 (this study). The GenBank accession numbers of these proteins are AY117395, AY117394, AY117393, AY117392, AY117391, AB094996, AB094997, AB094998, AB094999, AB095000, AB095001, AB095002, AB095003, and E02521), respectively [9, 23, 24]

Total mRNA was isolated from leucocytes of a domestic cat, and a FeIFN clone was isolated by PCR using primers designed from the sequences of FeIFN 7 and FeIFN 8. The open reading frame of the FeIFN clone consisted of 567 base pairs, predicted to encode a 189 amino acid protein with a molecular mass of 21.1 kDa (Fig. 1). An alignment with reported sequences (Fig. 1) showed that the amino acid sequence of this FeIFN clone was very similar to those of other FeIFNs, including type α subtypes 1–14 and type ω. Although subtypes α5, α7, and ω contained a five amino acid insertion, this clone did not (Fig. 2). The clone had a predicted signal peptide sequence at amino acids 1–23 (underlined) and a consensus site for asparagine-linked glycosylation (double underlined). The FeIFN is almost identical to α3, 9, 13, and 14 with the exception of four amino acid residues. Based on its amino acid sequence, the gene encoding this clone was defined as FeIFN-α15.

### Purification of rFeIFN-α15 from cocoons

**Fig. 3:**
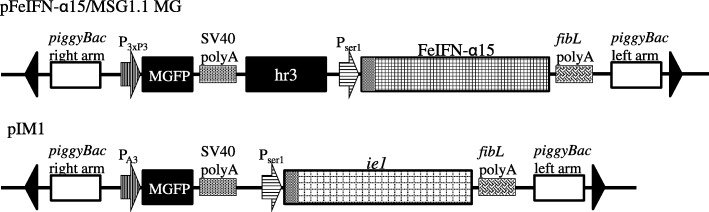
Structures of the transformation vectors pFeIFN-α15/MSG1.1 MG and pIM1. Each vector consisted of expression units for a selection marker and the recombinant protein, located between the right and left arms of *piggyBac*. The selection marker *MGFP* was placed between the 3xP3 promoter (P_3xP3_) and the SV40 polyA signal sequence (SV40 polyA) and between the *B. mori* actin promoter (P_A3_) and SV40 polyA in pFeIFNα-15/MSG1.1MG and pIM1 vectors, respectively. The cDNA of FeIFN-α15 on pFeIFN-α15/MSG1.1 MG was placed between the ser1 promoter (P_ser1_) and the fibroin L-chain polyA signal sequence (*fibL* polyA). The BmNPV hr3 enhancer was located upstream of P_ser1_, and the IE1 trans activator gene (*ie1*) on pIM1 was placed between P_ser1_ and *fibL* polyA

**Fig. 4:**
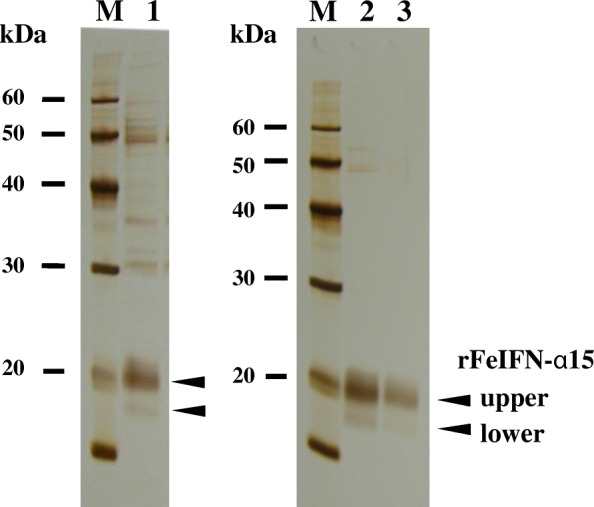
Extraction and purification of rFeIFN-α15 from cocoons. The crude extract obtained from the solubilisation of cocoon proteins with PBS (lane 1), the eluent from the Blue Sepharose™ column during the first step (lane 2), and the protein further purified on the ceramic hydroxyapatite column as the second step (lane 3) were analysed by SDS-PAGE followed by silver staining. The protein marker is shown as M

Using transformation vectors (Fig. 3), rFeIFN-α15 was expressed in the cocoons of transgenic silkworms. The resulting rFeIFN-α15 protein in the cocoons was dissolved in phosphate-buffered saline (PBS) and purified by two steps of column chromatography (Table 1). Starting with approximately 100 mg cocoons, 411 μg of crude proteins were extracted, including 267 μg rFeIFN-α15, making the latter 65% pure, as determined by silver staining of SDS-PAGE gels. The crude extracts were chromatographed on a Blue Sepharose column, yielding 93% pure rFeIFN-α15; this was followed by chromatography on a ceramic hydroxyapatite column, yielding 67 μg of 99% pure rFeIFN-α15 (Table 1 and Fig. 4). Electrophoresis of the purified rFeIFN-α15 on SDS-PAGE gels followed by silver staining showed two main bands, a lower band at less than 20 kDa and an upper band at around 20 kDa, both close to the expected molecular weight of mature rFeIFN-α15 (18.9 kDa). The upper and lower protein bands are likely the glycosylated and non-glycosylated proteins, respectively. The net recovery of rFeIFN-α15 from the crude extract was calculated to be 25%.

**Table 1:** Purification profile of rFeIFN-α15 from 100 mg of cocoon

Purification Step	Total Protein (μg)	Amount of rFeIFN-α15(μg)	Purity of rFeIFN-α15(%)
Crude Extract	411	267	65
Blue Sepharose™	107	100	93
Ceramic Hydroxyapatite	68	67	99

The amounts (middle) and purities (right) of rFeIFN-α15 relative to total protein (left) during each purification step are described

### N-terminal amino acid sequence of rFeIFN-α15

The five N-terminal amino acids of purified rFeIFN were analysed by Edman degradation (Table 2). Because the cDNA sequence indicated that the N-terminal residue of mature FeIFN-α15 was cysteine, the first degradation cycle did not show any peak as expected. The second to fifth amino acids were found to be D, L, P, and Q, consistent with the deduced amino acid sequence from the cDNA. The signal sequence of this protein was likely processed correctly for secretion into the cocoons.

**Table 2:** N-terminal amino acid sequence of purified rFeIFN-α15

Cycle no.	Amino acid	pmol
1	ND	–
2	D	26.1
3	L	18.9
4	P	10.5
5	Q	15.3

Purified rFeIFN-α15 was separated on a SDS-PAGE gel, and its N-terminal amino acid sequence was determined by five cycles of automated Edman degradation. The N-terminal sequences of the two main bands on SDS-PAGE were determined. Results obtained from analysis of the larger, 20-kDa band are shown; results obtained with the < 20-kDa band were identical. ND: No amino acid was detected during the first cycle of degradation, suggesting that it was cysteine

### Glycosidase digestion of rFeIFN-α15

**Fig. 5:**
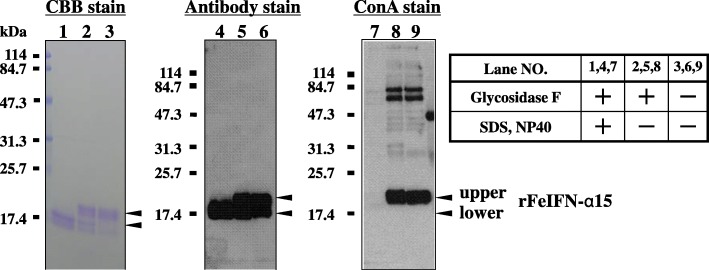
Glycosidase F digestion of rFeIFN-α15. rFeIFN-α15 was analysed by SDS-PAGE following glycosidase digestion in the presence (lanes 1, 4, and 7) or absence (lanes 2, 5, and 8) of SDS and NP-40 and in the absence of glycosidase (lanes 3, 6, and 9). One gel of each was stained with Coomassie Brilliant Blue (left panel; lanes 1, 2, and 3), whereas the others were treated with anti-FeIFN-α15 antibody (middle panel; lanes 4, 5, and 6) or Con A (right panel; lanes 7, 8, and 9)

Glycosylation of purified rFeIFN-α15 was assessed by blotting with an anti-IFN antibody and concanavalin A (Con A), which recognises mannose residues (Fig. 5). Treatment of the sample with glycosidase F in the presence of both SDS and NP-40 (Fig. 5, lane 1) resulted in an almost complete absence of the upper, 20-kDa protein band compared with the untreated control (Fig. 5, lane 3), probably because treatment with this enzyme removed all N-glycan residues and shifted its molecular weight to that of the lower, < 20-kDa protein band. This lower band was stained by both CBB (lane 1) and anti-FeIFN antibody (lane 4), but not by ConA (lane 7). In contrast, treatment with the enzyme in the absence of SDS and NP-40 or non-treatment with the enzyme resulted in both protein bands being stained by CBB (lanes 2 and 3) and anti-FeIFN antibody (lanes 5 and 6), although only the higher molecular weight band reacted with ConA (lanes 8 and 9). These results indicated that 20-kDa and < 20-kDa protein bands represented the N-glycosylated and non-glycosylated forms of rFeIFN-α15, respectively.

### Structure of N-glycans of rFeIFN-α15

Treatment of purified rFeIFN-α15 with pepsin followed by digestion with glycosidase A resulted in the release of N-glycans. The reducing ends of the N-glycans were labelled with 2-aminopyridine (PA), with each PA-oligosaccharide identified by column chromatography and mass spectrometric analysis. The N-glycan profile of rFeIFN-α15 is summarised in Table 3. Five PA-oligosaccharide fractions were obtained by column chromatography and were shown to be Man2Man3GlcNAc2-PA (M5), GlcNAcMan3GlcNAc2-PA (GNa), _GlcNAc_Man3GlcNAc2-PA (GNb), GlcNAc2Man3GlcNAc2-PA (GN2), and Man3GlcNAc2-PA (M3). The most abundant N-glycan, constituting 58.2% of the total, was M5 mannose-type, with about 30% being complex and hybrid types. About 10% of the total N-glycan was paucimannose-type (M3). There was no evidence of the existence of high-mannose-type (M6 or higher) or core-fucosylated glycans.

**Table 3:**
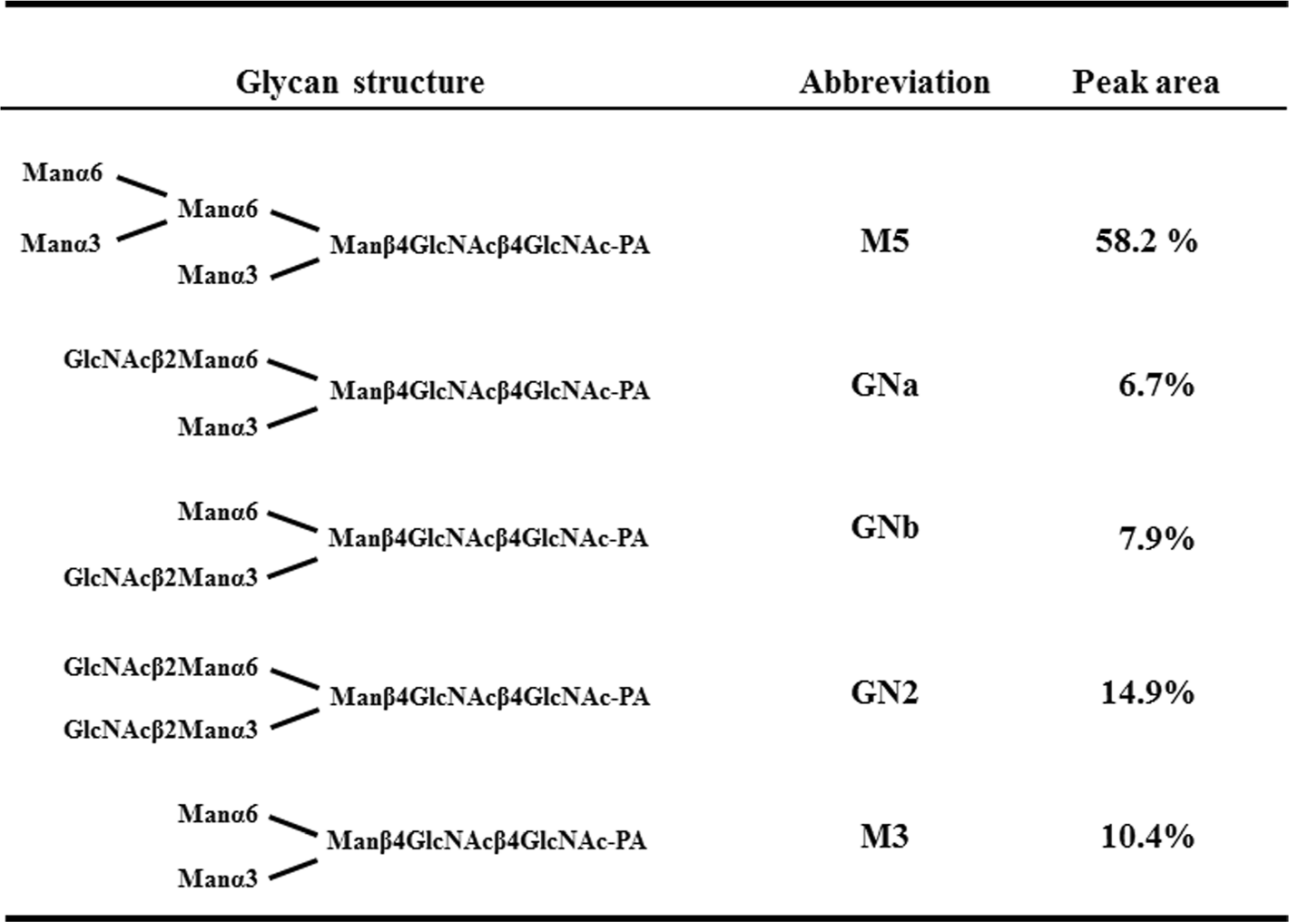
Structure of N-glycans attached to the rFeIFN-α15 produced by the transgenic silkworm system. The ratio (%) of each glycan was calculated from the corresponding peak area on the chromatogram obtained by HPLC

### In vitro anti-vesicular stomatitis virus activity

The titre of rFeIFN-α15 against vesicular stomatitis virus (VSV), measured using Fcwf-4 cells, was found to be 6.2 × 10^7^ units/mg in the crude extract and 2.6 × 10^8^ units/mg in the protein obtained after Blue Sepharose column chromatography (Table 4). It was reported that the anti-VSV titre of the baculovirus-produced rFeIFN-ω measured by using Fc9 cells was 2.4 × 10^8^ units/mg [14]. Although the feline cell lines used in the measurements differed, the rFeIFN-α15 and rFeIFN-ω are likely to possess VSV activities at similar levels.

**Table 4:** Anti-VSV activity of rFeIFN-α15 as measured using CPE assay

Sample	unit/mg
Crude extract	6.2 × 10^7^
Blue Sepharose™ column elute	2.6 × 10^8^

The inhibitory activity of rFeIFN-α against VSV infection of Fcwf-4 cells was determined by a 50% reduction in CPE. Commercially available rFeIFN-ω was used as a standard

## Discussion

To date, 14 subtypes of FeIFN-α have been reported [24, 25]. In the present study, we cloned the fifteenth subtype of feline interferon α gene, named FeIFN-α15. We compared the protein product of this gene with rFeIFN-ω (NCBI Number: E02521), classified as a FeIFN-α [24–26], produced in silkworm larvae using the baculovirus expression system. Expression of rFeIFN-ω from its original signal sequence in silkworm larvae resulted in processing of the N-terminal peptide at a site different from the natural version [27]. Because the peptide sequence of human calreticulin has been reported to be processed at the correct site in MSG [28], we used the signal sequence of human calreticulin for the expression of FeIFN-α15 in this study. We found that this signal sequence was processed at the correct site and that the mature form of rFeIFN-α15, carrying its natural N-terminal sequence, was secreted into cocoons.

The N-glycan structure of rFeIFN-α15 was assessed using glycosidase F, an enzyme that can cleave N-glycan linkages in the presence of both SDS and NP-40 and when the α 1,3 fucose is not attached to the core N-acetylglucosamine (GlcNAc). The results obtained indicated that N-glycans attached to the rFeIFN-α15 did not contain core α 1,3 fucosyl residues. Furthermore, comparison of the N-glycan structures of the rFeIFN-α15 produced by the transgenic silkworm system during expression in MSG and of the ω type produced by the baculovirus expression system in entire silkworm larvae [10] showed noticeable differences between them. The N-glycan profile of rFeIFN-α15 by the transgenic silkworm system in MSG consisted of about 30% hybrid- and complex-types, 58% M5, and 10% paucimannose-type. No high-mannose-type (M6 or higher) nor core α 1,3 fucosylated glycans were detected. This profile differs markedly from the typical insect profile. Although the precise N-glycan structures of rFeIFN-ω have not been determined, the sugar chains were reported to consist of mannose, GlcNAc, and fucose, in a 2: 2: 1 ratio [14]. These data suggest that the N-glycans of rFeIFN-ω had a typical insect-type profile, with FM2 (Man2GlcNAc2Fuc) thought to be a main N-glycan [14]. The N-glycan structures of recombinant mouse IFN-β produced by the baculovirus expression system were reported to consist of 20% high-mannose-type (more than M6), 51% paucimannose-type, and 12% core α 1,3-fucosylated glycans [29]. Thus, the N-glycan pattern of rFeIFN-α15 synthesised in silk glands in this study differed from those of previously reported rIFNs synthesised in the baculovirus expression system, in which recombinant proteins were expressed in baculovirus-infected organs, such as fat bodies or haemolymph except silk glands.

These findings and those regarding IgG production in MSG [20] suggest that the mechanisms of N-glycosylation, especially core α 1,3 fucosylation, in MSG differ from those in other organs. The silk gland is specialised to produce proteins required for weaving cocoons, and MSG is involved in the production of sericin, which covers the fibroin core. This specific functional feature of MSG may be related to the specificity of N-glycan structure. Although detailed analysis of the glycosylation mechanism of MSG is necessary, it is thought that the specificity of the N-glycan structure in MSG provides a significant benefit with regard to the production of safe recombinant proteins with insects.

The anti-VSV titres of rFeIFN-α7 and ω, both of which had a five amino acid insertion, were approximately 10 times higher than the titres of rFeIFN-α8 and -α10 without the insertion [30]. As these rIFNs were produced in *E. coli* without any N-glycans on them, the high anti-VSV titres of rFeIFN-α7 and -ω have been attributed to the five amino acid insertion. However, our study showed that the anti-VSV titre of rFeIFN-α15 (without the insertion) was comparable to that of rFeIFN-ω (with the insertion), suggesting that anti-VSV activity may be affected by both the N-glycan profile and amino acid insertions.

In this study, the anti-viral activity of the recombinant rFeIFN-α15 was evaluated by in vitro assay with a single kind of virus, VSV. To demonstrate the clinical usability of the rFeIFN-α15, in vivo studies for anti-viral activities to several kinds of feline infective viruses should be evaluated.

## Conclusion

This study showed that the rFeIFN-α15 produced by the transgenic silkworm system had the N-terminal amino acid sequence identical to the natural one and had no core α 1,3 fucosyl residues in its N-glycans. Additionally, the anti-VSV activity of the rFeIFN-α15 assessed in vitro was comparable to that of rFeIFN-ω produced by the baculovirus expression system in silkworms. These results indicate that rFeIFN-α15 may be useful in veterinary medication, but further studies including an assessment of in vivo anti-viral activity would be required to verify its availability.

## Methods

### Experimental animals

*B. mori* strain w1-pnd was obtained from the National Institute of Agrobiological Science (Tsukuba, Japan). The larvae were reared at 25 °C on an artificial diet (Silk MatePM; Nosan Corp, Kanagawa, Japan). Feline blood was obtained from a Japanese domestic cat acting as a donor for blood transfusion. Fcwf-4 cells, the cell line derived from feline embryo fibroblasts, were purchased from the ATCC (ATCC no. CRL-2787).

### Cloning of FeIFN-α and vector construction

Leukocytes were isolated from feline blood, and total RNA was extracted from the cells using TRIzol® LS Reagent (Thermo Fisher Scientific K.K., Yokohama, Japan). IFN-α cDNA was synthesised using a One-Step RT-PCR Kit (Invitrogen, Carlsbad, USA) with the two amplification primers, 5- ATGGCGCTGCCCTCTTCCTTCTTGG-3′ (forward) and 5’-TTTCTCGCTCCTTAATCTTTTCTGC-3′ (reversed) derived from the common sequences of IFNA7 and IFNA8 (GenBank Accession Nos. NM_001009197 and NM_001009193, respectively). The cDNA fragments obtained by reverse transcription polymerase chain reaction (RT-PCR) were sequenced. The cDNAs encoding the open reading frames of full-length FeIFN-α (IFNα family) were cloned. FeIFN-α15, a novel type of FeIFN-α, was also isolated (GenBank Accession No. AB608218). The predicted signal sequence region of FeIFN-α15 was replaced with that of human calreticulin, as this signal sequence has been reported to be processed at the correct site in MSG [28]. The PCR product amplified using the following primer sets was inserted into a cloning vector. The 5′-untranslated region (UTR) sequence of *Bombyx mori* nuclear polyhedrosis virus (BmNPV) polyhedrin [31, 32] was inserted upstream of the cDNAs by this PCR amplification using the primers 5’-CAC**CCCCGGG**AAGTATTTTACTGTTTTCGTAACAGTTTTGTAATAAAAAAACCTATAAATATGCTGCTATCCGTGCCGTTGCTGCTCGGCCTCCTCGGCCTGGCCGTCGCCTGTGACCTGCCTCAGACCCACG-3′ (forward) and 5’-**CCCGGG**TTATTTCTCGCTCCTTAATCTTTTCTGC-3′ (reverse), where the bold letters indicate a restriction site for the enzyme *Sma*I. The resulting fragment was inserted into the pENTR_D-TOPO cloning vector (Invitrogen), released from the vector by digesting with *Sma*I, and inserted into the plasmid pMSG1.1MG to yield pFeIFN-α15/MSG1.1MG for generation of transgenic silkworms [28, 32].

### Generation of a TG silkworm line expressing rFeIFN-α15

pFeIFN-α15/MSG1.1MG was injected, along with the helper vector pHA3PIG [33], into pre-blastoderm silkworm embryos as described [33]. The hatched G0 larvae were reared to moths at 25 °C. G1 embryos were generated by mating siblings or w1-pnd and screened for ocular expression of monster green fluorescent protein (MGFP), with those showing positive expression defined as transgenic silkworms bearing the FeIFN-α15 gene. These transgenic silkworms were subsequently mated with transgenic silkworms expressing the trans-activator IE1 in the MSG, as described [28] (Fig. 3), yielding transgenic silkworms expressing higher levels of rFeIFN-α15.

### Extraction and purification of rFeIFN-α15 from cocoons

Sheared cocoons were immersed in PBS, pH 7.4, at 20 mg/ml and maintained at 4 °C for 24 h. The crude extract was centrifuged, with the supernatant dialysed against 10 mM sodium phosphate buffer, pH 7.4, 150 mM NaCl and applied to a Blue Sepharose™ column (HiTrap™ Blue, GE Healthcare, Tokyo, Japan). rFeIFN was eluted with a linear gradient concentration of buffer containing 1.0 M NaCl. The fractionated eluent from the Blue Sepharose™ column was analysed by SDS-PAGE. Fractions containing rFeIFN-α15 were collected, dialysed against 5 mM potassium phosphate buffer, pH 7.0, 100 mM NaCl, and loaded onto a ceramic hydroxyapatite column. rFeIFN was eluted with a linear gradient concentration of buffer containing 500 mM NaCl. The purified rFeIFN-α15 was used for various assays except for N-glycan analysis. For N-glycan analysis of rFeIFN-α15, the protein eluted from the Blue Sepharose™ column was dialysed against 5 mM potassium phosphate buffer, pH 7.0, 150 mM NaCl and purified on a Superdex 75 gel filtration column (GE Healthcare).

Total protein content in the extract and purified materials was quantified using a micro bicinchoninic acid protein assay kit (Thermo Fisher Scientific K.K.). The ratio of rFeIFN-α15 to total protein was analysed by SDS-PAGE and silver staining, with the gel images scanned with ChemiDoc XRS (Bio-Rad, Tokyo, Japan). The amount of purified rFeIFN-α15 was calculated from the total protein content and the ratio of rFeIFN-α15 (Table 1).

### N-terminal amino acid sequence of rFeIFN-α15

The N-terminal sequence of purified rFeIFN-α15 was determined with a protein sequencer, HP G1005A (Agilent Technologies International Japan, Tokyo, Japan), using a standard Edman degradation protocol.

### Glycosidase digestion and blotting assays

Purified rFeIFN-α15 was digested with glycosidase F (Takara Bio, Shiga, Japan) at 37 °C for 17 h in the presence or absence of 0.5% SDS and 1.0% NP-40. The samples were separated on SDS-PAGE, followed by blotting to Immobilon-P membranes (Merck Millipore, Tokyo, Japan). For immunoblotting, the membranes were incubated with primary anti-FeIFN-α15 polyclonal rabbit antibody, generated by us, and secondary horseradish peroxidase (HRP)-conjugated anti-rabbit goat IgG (Sigma-Aldrich, Tokyo, Japan), with HRP detected with chemiluminescent reagents (ECL Western Blotting Detection System) (GE Healthcare). For lectin-blotting, the membranes were treated with biotinylated Con A (Seikagaku Corporation, Tokyo, Japan) and the resulting biotin residues reacted with streptavidin HRP conjugate (Sigma-Aldrich). HRP was detected with 3,3-diaminobenzidine (Wako Chemicals, Tokyo, Japan).

### N-glycan analysis of rFeIFN-α15

N-glycan analysis of purified rFeIFN-α15, including glycosidase digestion of the protein, pyridylamination of the released glycans, and separation of PA-oligosaccharides by column chromatography, was performed as previously described [34].

### Mass spectrometric analyses of PA-oligosaccharides

PA-oligosaccharides were subjected to matrix-assisted laser desorption/ionisation time-of-flight mass spectrometric (MALDI-TOF MS) analysis, as previously described [35].

### Antiviral activity assay of rFeIFN-α15

The antiviral activity of rFeIFN-α15 was determined in vitro using a standard VSV assay [36]. Fcwf-4 cells were inoculated into 96-well plates at a density of 2 × 10^4^ cells per well and cultured in Dulbecco’s Modified Eagle’s medium supplemented with 5% foetal bovine serum (FBS) (Sigma-Aldrich) and 1% penicillin and streptomycin (Thermo Fisher Scientific K.K.) for 16 h in an atmosphere containing 5% CO_2_. The crude extract or proteins eluted from the Blue Sepharose™ column were serially diluted with the media supplemented with 1% FBS, and each 100 μL of the media was added into the Fcwf-4 cell-containing wells. They were cultured for 20 h. Intercat1 (Toray Industries, Tokyo, Japan), a commercially available rFeIFN-ω, was used as a positive control. After each supernatant was removed, the plates were further incubated for 48 h at 37 °C, followed by infection with 100 μL of VSV (VR-158) (Summit Pharmaceuticals International Corporation, Tokyo, Japan) at a concentration of 1000 TC ID_50_/ml. When the cytopathic effect (CPE) was greater than 95% of the control cells (without FeIFN), all the plates were stirred gently, and the cells were washed three times with PBS, fixed in 10% (*v*/v) formalin and stained with 0.1% (*w*/*v*) crystal violet. The stained cells were washed with deionised water and allowed to dry. The crystal violet was eluted with 100 μL of methanol, and the absorbance of each well was measured at 595 nm with a micro-titre plate reader (Spectra Max 250; Molecular Devices Japan, Tokyo, Japan). Titres of the samples were corrected by the titres of Intercat1.

## Abbreviations

BmNPV: *Bombyx mori* nuclear polyhedrosis virus
Con A: Concanavalin A
CPE: Cytopathic effect
FBS: Foetal bovine serum
FeIFN: Feline interferon
fibL polyA: fibroin L-chain polyA signal sequence
FM2: Man2GlcNAc2Fuc
GlcNAc: N-acetylglucosamine
GN2: GlcNAc2Man3GlcNAc2-PA
GNa: GlcNAcMan3GlcNAc2-PA
GNb: _GlcNAc_Man3GlcNAc2-PA
HPLC: High-performance liquid chromatography
hr3: hr3 enhancer
ie1: IE1 trans activator gene
M3: Man3GlcNAc2-PA
M5: Man2Man3GlcNAc2-PA
MALDI-TOF MS: Matrix-assisted laser desorption/ionisation time-of-flight mass spectrometric
MGFP: Monster green fluorescent protein
MSG: Middle silk gland
P3xP3: 3xP3 promoter
PA: aminopyridine
PAGE: Polyacrylamide gel electrophoresis
PBS: Phosphate-buffered saline
PCR: Polymerase chain reaction
Pser1: Ser1 promoter
SDS: Sodium dodecyl sulphate
SV40 polyA: SV40 polyA signal sequence
TG: Transgenic
UTR: Untranslated region
VSV: Vesicular stomatitis virus

## References

[1] Geisow MJ (1992). Glycoprotein glycans--roles and controls. Trends Biotechnol.

[2] Goldwasser E, Kung CK, Eliason J (1974). On the mechanism of erythropoietin-induced differentiation. 13. The role of sialic acid in erythropoietin action. J Biol Chem.

[3] Dordal MS, Wang FF, Goldwasser E (1985). The role of carbohydrate in erythropoietin action. Endocrinology.

[4] Dube S, Fisher JW, Powell JS (1988). Glycosylation at specific sites of erythropoietin is essential for biosynthesis, secretion, and biological function. J Biol Chem.

[5] Luckow VA, Summers MD (1989). High level expression of nonfused foreign genes with Autographa californica nuclear polyhedrosis virus expression vectors. Virology.

[6] Smith GE, Summers MD, Fraser MJ (1983). Production of human beta interferon in insect cells infected with a baculovirus expression vector. Mol Cell Biol.

[7] Maeda S, Kawai T, Obinata M, Fujiwara H, Horiuchi T, Saeki Y, Sato Y, Furusawa M (1985). Production of human alpha-interferon in silkworm using a baculovirus vector. Nature.

[8] Nagaya H, Kanaya T, Kaki H, Tobita Y, Takahashi M, Takahashi H, Yokomizo Y, Inumaru S (2004). Establishment of a large-scale purification procedure for purified recombinant bovine interferon-tau produced by a silkworm-baculovirus gene expression system. J Vet Med Sci.

[9] Nakamura N, Sudo T, Matsuda S, Yanai A (1992). Molecular cloning of feline interferon cDNA by direct expression. Biosci Biotechnol Biochem.

[10] Sakurai T, Ueda Y, Sato M, Yanai A (1992). Feline interferon production in silkworm by recombinant baculovirus. J Vet Med Sci.

[11] Ueda Y, Sakurai T, Kasama K, Satoh Y, Atsumi K, Hanawa S, Uchino T, Yanai A (1993). Pharmacokinetic properties of recombinant feline interferon and its stimulatory effect on 2′,5′-oligoadenylate synthetase activity in the cat. J Vet Med Sci.

[12] Minagawa T, Ishiwata K, Kajimoto T (1999). Feline interferon-omega treatment on canine parvovirus infection. Vet Microbiol.

[13] Tretter V, Altmann F, Kubelka V, Marz L, Becker WM (1993). Fucose alpha 1,3-linked to the core region of glycoprotein N-glycans creates an important epitope for IgE from honeybee venom allergic individuals. Int Arch Allergy Immunol.

[14] Ueda Y, Sakurai T, Mizuno Y, Hujii Y, Yanai A (1996). Characterization of oligosaccharides associated with feline interferon ω produced in recombinant baculovirus-infected Bombyx mori. J Seric Sci Jpn.

[15] Minagawa S, Sekiguchi S, Nakaso Y, Tomita M, Takahisa M, Yasuda H (2015). Identification of Core alpha 1,3-Fucosyltransferase gene from silkworm: an insect popularly used to express mammalian proteins. J Insect Sci.

[16] Wilson IB, Harthill JE, Mullin NP, Ashford DA, Altmann F (1998). Core alpha1,3-fucose is a key part of the epitope recognized by antibodies reacting against plant N-linked oligosaccharides and is present in a wide variety of plant extracts. Glycobiology.

[17] Tomita M, Munetsuna H, Sato T, Adachi T, Hino R, Hayashi M, Shimizu K, Nakamura N, Tamura T, Yoshizato K (2003). Transgenic silkworms produce recombinant human type III procollagen in cocoons. Nat Biotechnol.

[18] Tomita M, Hino R, Ogawa S, Iizuka M, Adachi T, Shimizu K, Sotoshiro H, Yoshizato K (2007). A germline transgenic silkworm that secretes recombinant proteins in the sericin layer of cocoon. Transgenic Res.

[19] Ogawa S, Tomita M, Shimizu K, Yoshizato K (2007). Generation of a transgenic silkworm that secretes recombinant proteins in the sericin layer of cocoon: production of recombinant human serum albumin. J Biotechnol.

[20] Iizuka M, Ogawa S, Takeuchi A, Nakakita S, Kubo Y, Miyawaki Y, Hirabayashi J, Tomita M (2009). Production of a recombinant mouse monoclonal antibody in transgenic silkworm cocoons. FEBS J.

[21] Chen WY, Shen QX, Bahl OP (1991). Carbohydrate variant of the recombinant beta-subunit of human choriogonadotropin expressed in baculovirus expression system. J Biol Chem.

[22] Kuroda K, Geyer H, Geyer R, Doerfler W, Klenk HD (1990). The oligosaccharides of influenza virus hemagglutinin expressed in insect cells by a baculovirus vector. Virology.

[23] Shi X, Jarvis DL (2007). Protein N-glycosylation in the baculovirus-insect cell system. Curr Drug Targets.

[24] Wonderling R, Powell T, Baldwin S, Morales T, Snyder S, Keiser K, Hunter S, Best E, McDermott MJ, Milhausen M (2002). Cloning, expression, purification, and biological activity of five feline type I interferons. Vet Immunol Immunopathol.

[25] Nagai A, Taira O, Ishikawa M, Hiramatsu K, Hohdatsu T, Koyama H, Arai S, Sato H, Nakano K, Maehara N (2004). Cloning of cDNAs encoding multiple subtypes of feline interferon-alpha from the feline epitherial cell line. J Vet Med Sci.

[26] Yang LM, Xue QH, Sun L, Zhu YP, Liu WJ (2007). Cloning and characterization of a novel feline IFN-omega. J Interf Cytokine Res.

[27] Ueda Y, Sakurai T, Yanai A (1993). Homogeneous production of feline interferon in silkworm by replacing single amino acid code in signal peptide region in recombinant baculovirus and characterization of the product. J Vet Med Sci.

[28] Adachi T, Wang X, Murata T, Obara M, Akutsu H, Machida M, Umezawa A, Tomita M (2010). Production of a non-triple helical collagen alpha chain in transgenic silkworms and its evaluation as a gelatin substitute for cell culture. Biotechnol Bioeng.

[29] Misaki R, Nagaya H, Fujiyama K, Yanagihara I, Honda T, Seki T (2003). N-linked glycan structures of mouse interferon-beta produced by Bombyx mori larvae. Biochem Biophys Res Commun.

[30] Taira O, Suzuki M, Takeuchi Y, Aramaki Y, Sakurai I, Watanabe T, Motokawa K, Arai S, Sato H, Maehara N (2005). Expression of feline interferon-alpha subtypes in Esherichia coli, and their antiviral activity and animal species specificity. J Vet Med Sci.

[31] Iatrou K, Ito K, Witkiewicz H (1985). Polyhedrin gene of Bombyx mori nuclear polyhedrosis virus. J Virol.

[32] Iizuka M, Tomita M, Shimizu K, Kikuchi Y, Yoshizato K (2008). Translational enhancement of recombinant protein synthesis in transgenic silkworms by a 5′-untranslated region of polyhedrin gene of Bombyx mori Nucleopolyhedrovirus. J Biosci Bioeng.

[33] Tamura T, Thibert C, Royer C, Kanda T, Abraham E, Kamba M, Komoto N, Thomas JL, Mauchamp B, Chavancy G (2000). Germline transformation of the silkworm Bombyx mori L. using a piggyBac transposon-derived vector. Nat Biotechnol.

[34] Nakagawa H, Kawamura Y, Kato K, Shimada I, Arata Y, Takahashi N (1995). Identification of neutral and sialyl N-linked oligosaccharide structures from human serum glycoproteins using three kinds of high-performance liquid chromatography. Anal Biochem.

[35] Yagi H, Takahashi N, Yamaguchi Y, Kimura N, Uchimura K, Kannagi R, Kato K (2005). Development of structural analysis of sulfated N-glycans by multidimensional high performance liquid chromatography mapping methods. Glycobiology.

[36] Familletti PC, Rubinstein S, Pestka S (1981). A convenient and rapid cytopathic effect inhibition assay for interferon. Methods Enzymol.

